# Circulating fibroblast growth factor-21 in coronary artery disease: a systematic review and meta-analysis

**DOI:** 10.1007/s11845-026-04472-2

**Published:** 2026-06-03

**Authors:** Feryal Shawa, Fatma Izzeddin, Gulsah Koc

**Affiliations:** 1 Institute of Graduate Studies, Molecular Medicine Program, Istanbul Aydin University, Istanbul, Turkey; 2 Department of Medical Biology, Faculty of Medicine, Istanbul Aydin University, Istanbul, Turkey

**Keywords:** Fibroblast growth factor 21, Coronary artery disease, Acute coronary syndrome, Myocardial infarction, Biomarker, Meta-analysis

## Abstract

**Background:**

Fibroblast growth factor-21 (FGF21) is a metabolic regulator involved in glucose and lipid homeostasis and has emerged as a potential biomarker in cardiometabolic diseases. The relationship between circulating FGF21 levels and coronary artery disease (CAD) remains unclear. We performed a systematic review and meta-analysis to clarify this association and assess its clinical relevance.

**Methods:**

PubMed, Web of Science, and Scopus were searched for observational studies reporting circulating FGF21 levels in adult CAD patients. Standardized mean differences (SMDs) with 95% confidence intervals were calculated using a random-effects model. Subgroup and meta-regression analyses were conducted to explore heterogeneity.

**Results:**

Fourteen studies including 2963 CAD patients were analyzed. Circulating FGF21 levels were significantly higher in CAD patients than in controls (SMD = 0.90; 95% CI: 0.26–1.54; *p* = 0.0056). In acute CAD, the increase was more pronounced (SMD = 1.70; 95% CI: 0.20–3.21). Elevated FGF21 persisted even when diabetes prevalence was low (SMD = 1.50; 95% CI: 0.15–2.84). Meta-regression identified BMI and HDL as independent contributors to inter-study variability. Sensitivity analyses confirmed robustness, and no strong evidence of publication bias was detected.

**Conclusions:**

Circulating FGF21 is elevated in CAD, primarily reflecting metabolic disturbances—particularly adiposity and dyslipidemia—rather than diabetes or CAD itself. Levels tend to be higher during acute events, suggesting that FGF21 may serve as a marker of metabolic stress and lipid abnormalities rather than a direct diagnostic biomarker.

**Supplementary Information:**

The online version contains supplementary material available at https://doi.org/10.1007/s11845-026-04472-2.

## Introduction

Coronary artery disease (CAD) is the most prevalent form of cardiovascular disease and remains one of the leading causes of mortality from non-communicable diseases worldwide, particularly in high-income regions [1]. As a chronic inflammatory atherosclerotic disorder, CAD may clinically manifest as stable angina pectoris (SAP), unstable angina pectoris (UAP), myocardial infarction (MI), or sudden cardiac death [2].

Given the heterogeneous clinical course and diagnosis of CAD and related cardiovascular conditions, the identification of novel biomarkers for early diagnosis, risk stratification, and prognostic evaluation has gained increasing attention [3]. Fibroblast growth factor 21 (FGF21), a metabolic regulator involved in glucose and lipid homeostasis with endocrine properties, has emerged as a potential biomarker and therapeutic target in cardiometabolic diseases. Beyond its metabolic functions, FGF21 exerts direct biological effects on the cardiovascular system and may protect against tissue injury associated with metabolic dysregulation [3, 4].

Large-scale epidemiological studies conducted in human populations have demonstrated that elevated serum FGF21 levels are significantly associated with obesity, impaired glucose tolerance, and type 2 diabetes mellitus (T2DM) [5, 6]. In addition, several studies have reported increased serum FGF21 levels in patients with CAD [7–9]. However, findings regarding the diagnostic value of FGF21 in CAD remain inconsistent; while some studies suggest that higher FGF21 levels are associated with disease severity and adverse clinical outcomes, others have failed to confirm this relationship [10]. Consequently, the available evidence is characterized by inconsistency and substantial methodological and clinical heterogeneity [11].

Most existing studies are limited by relatively small sample sizes, heterogeneous patient populations, variations in measurement techniques, and differences in defined clinical endpoints, which restrict the comparability and generalizability of their findings [11–13]. Moreover, a comprehensive quantitative synthesis evaluating the association between circulating FGF21 levels and clinical outcomes in CAD is currently lacking, highlighting the need to integrate the available evidence through a systematic review and meta-analysis.

Therefore, the aim of this systematic review and meta-analysis was to quantitatively evaluate the association between circulating FGF21 levels and coronary artery disease, and to determine their clinical and diagnostic significance by synthesizing the available evidence.

## Materials and methods

### Data sources and search strategy

This meta-analysis was conducted in accordance with the Preferred Reporting Items for Systematic Reviews and Meta-Analyses (PRISMA) guidelines [14]. A systematic and comprehensive literature search was performed in PubMed, Web of Science, and Scopus databases on 20 January 2026, with no language restrictions, to identify studies reporting serum FGF21 levels in patients with CAD. The search strategy was developed based on the PICOS (Population, Intervention/Exposure, Comparison, Outcomes, and Study design) principle. Specifically, the search terms combined those for the exposure, including **“**fibroblast growth factor 21” or FGF21, with terms describing the study population and outcomes, such as “coronary artery disease,” “ischemic heart disease,” “acute coronary syndrome,” or “myocardial infarction.”

### Inclusion and exclusion criteria

Inclusion criteria:

1. Observational human studies (cohort, case-control, or cross-sectional) reporting circulating FGF21 protein in adult patients (≥ 18 years) diagnosed with CAD, confirmed by clinical, biochemical, and/or imaging criteria.
2. CAD was defined as either stable coronary artery disease (including stable angina with angiographically confirmed stenosis) or acute coronary syndrome (ACS), comprising unstable angina, NSTEMI, and STEMI.
3. Circulating FGF21 levels (serum or plasma) measured, with clearly reported units (e.g., pg/mL, ng/mL).
4. Inclusion of CAD-free control group.
5. Reported differences in FGF21 levels between CAD patients and controls, using mean ± standard deviation or median (IQR), allowing calculation of standardized mean differences (SMD).
6. Full-text articles accessible.

Exclusion criteria:

1. Patient populations beyond CAD, encompassing heart failure, stroke, and other cardiovascular disorders.
2. Pediatric or obstetric populations.
3. Incomplete, inconsistent, or unverifiable data.
4. Reviews, meta-analyses, case reports, editorials, conference abstracts, or letters.
5. Studies conducted on animals or in vitro.
6. Duplicate publications from the same population.

### Data extraction

All identified records were imported into Zotero reference management software, and duplicate entries were removed prior to screening. Study selection was conducted in two phases. In the first phase, titles and abstracts were screened to identify potentially eligible studies. In the second phase, the full texts of relevant articles were retrieved and assessed for eligibility based on the predefined inclusion and exclusion criteria. Two reviewers (FS and FI) extracted data from the included articles independently to reduce the selective bias and enhance credibility. Data extraction was performed using a double-blind approach, with any discrepancies resolved through consensus. In cases where consensus could not be achieved, a 3rd researcher (GK) was consulted to make the final decision.

The following data were extracted from each study: first author, publication year, country, study design, CAD type, sample size, demographic characteristics (age and sex), and circulating FGF21 levels in CAD and control groups. In addition, cardiometabolic characteristics—including the proportion of participants with diabetes, LDL cholesterol, HDL cholesterol, body mass index (BMI), and fasting glucose levels—were collected for both CAD and control groups. These variables were subsequently incorporated into subgroup and meta-regression analyses to explore potential sources of between-study heterogeneity.

### Quality assessment

Quality assessment was independently performed by two reviewers (FS and FI). Discrepancies were resolved by discussion with a third reviewer (GK). The methodological quality of cohort and case–control studies were assessed by using the Newcastle–Ottawa Quality Assessment Scale (NOS) [15]. Based on NOS scores, studies were categorized as high quality (≥ 7 points), moderate quality (5–6 points), or low quality (≤ 4 points). Cross-sectional studies were appraised using the Joanna Briggs Institute (JBI) Critical Appraisal Checklist for Analytical Cross-Sectional Studies [16]. Detailed results of the quality assessment are provided in Table S1.

### Data analysis

The meta-analysis was conducted using R Studio (version 2026.01.0 + 392) with relevant meta-analysis packages. Statistical heterogeneity across studies was assessed using the Chi-squared (χ²) test and the I² statistic. A p-value < 0.05 or an I² value > 50% was considered indicative of significant heterogeneity. In the presence of significant heterogeneity, a random-effects model was applied. The overall effect size was expressed as a standardized mean difference (SMD) with corresponding 95% confidence intervals (CIs). Statistical significance was determined using the Z-test, with *p* < 0.05 considered statistically significant.

To explore potential sources of heterogeneity, subgroup analyses were conducted according to CAD classification (acute vs. stable) and the proportion of participants with diabetes (high vs. low). Additionally, univariable meta-regression analyses were performed to evaluate whether between-group differences in cardiometabolic parameters—including LDL cholesterol, HDL cholesterol, body mass index (BMI), and fasting glucose levels—were associated with the pooled effect size. Furthermore, a multivariable meta-regression model incorporating fasting glucose, BMI, and HDL cholesterol simultaneously was constructed to assess their independent contributions to between-study heterogeneity.

Sensitivity analysis was carried out using a leave-one-out approach to determine whether the pooled SMD was substantially influenced by any single study. Publication bias was evaluated through visual inspection of funnel plots, and funnel plot asymmetry was further assessed using Egger’s linear regression test and Begg’s rank correlation test. For studies reporting data as median and interquartile range (IQR), values were converted to mean ± standard deviation (SD) using the method described by Wan et al. [17]. Calculations were performed using the Meta-Analysis Accelerator web-based tool.

In the study by Koprulu et al. [18], patients were categorized into subgroups representing the same underlying clinical condition, and these subgroups were combined prior to analysis. Pooled means and standard deviations for all continuous variables were calculated using weighted averaging methods based on sample size.

In the study by Cheng et al. [19], a single control group was used for multiple patient subgroup comparisons. To avoid unit-of-analysis errors, the control group sample size was proportionally divided across comparisons in accordance with recommendations from the Cochrane Handbook for Systematic Reviews of Interventions [20].

## Results

### Included studies and their characteristics

**Fig. 1:**
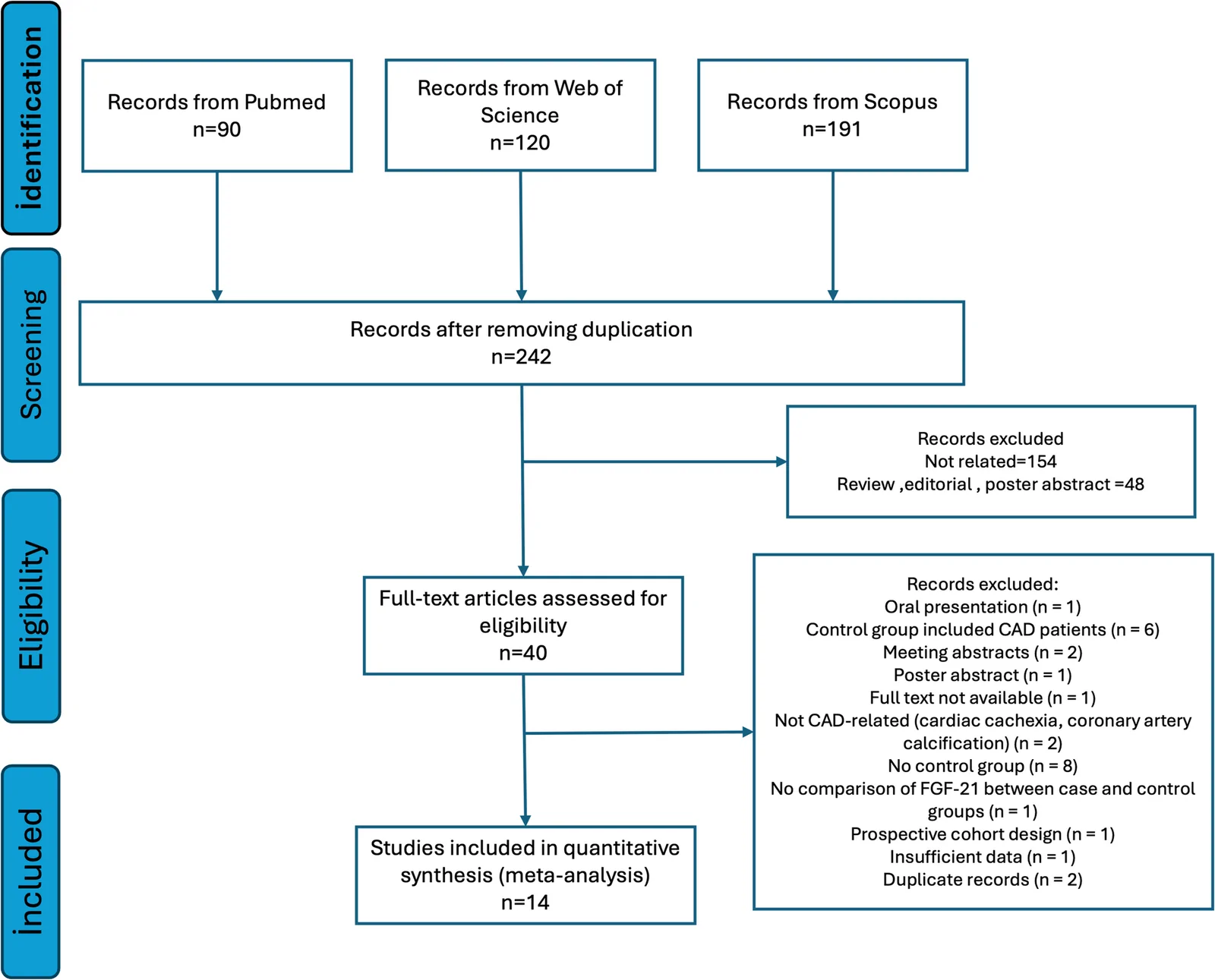
A flow diagram detailing the search strategy and study selection process for this systematic review and meta-analysis on circulating levels of FGF21 in patients with CAD

After conducting a comprehensive search, we identified 401 potentially relevant articles. Of these, 159 duplicates were removed, leaving 242 articles for title and abstract screening. Following this review, 202 articles were excluded. The remaining 40 articles underwent full-text assessment, and 14 studies [7, 8, 10, 12, 13, 18, 19, 21–27], comprising a total of 2963 participants, were ultimately included in the meta-analysis. The study selection process is illustrated in Fig. 1. Baseline characteristics of the included studies are summarized in Table 1. Detailed metabolic parameters, including BMI, HDL, LDL, fasting glucose concentrations, and the proportion of participants with diabetes in both patient and control groups, are detailed in Supplementary Table 2.

**Table 1:** Baseline characteristics of studies included in the meta-analysis

Study	Country	Study design	Disease	CAD group (n)	Control group (n)	Male (%)		Age (years)*		FGF21 concentration (pg/ml)	
						CAD	Control	CAD	Control	CAD	Control
Chen et al.	China	Case-control	CAD	252	153	69.7	30.3	64.3±9.6	62.0±9.7	334.4 (180.8–548.6)^#^	376.1(247.2–588.7)^#^
Zhang et al.	China	Prospective cohort	AMI	24	18	70.8	44.4	55±15	61±9	210 ± 150*	100 ± 60*
Qian et al.	China	Prospective cohort	ACS	304	96	66.1	38.5	64.54±10.29	55.75±8.27	361.98 (346.45–381.02)^#^	269.85 (257.92–326.58)^#^
Lin et al.	China	Cross-sectional	CHD (MI+SAP)	135	61	49.6	50.8	69±5.8	68.6±10.8	362.5 (210.6–661.9)^#^	131 (70.4–249.5)^#^
Koprulu et al.	Turkey	Case-control	STEMI	91	45	60.4	58	60.6±12.8	58.8±9.6	110.7 ± 46.0*	78.95 ± 13*
Chen et al.	China	Prospective cohort	AMI	183	55	84.7	60	63.6±10.9	66.9±9.5	143.8 (75.2–254.3)^#^	121 (57.1–179.6)^#^
Cheng et al.**	China	Case-control	SAP	60	27	54.55	38.18	60.67±9.51	59.38±9.18	4.85 ± 0.77*	4.54 ± 0.72*
			UAP	76	28	68.42	38.18	60.05±8.23	59.38±9.18	5.26 ± 0.87*	4.54 ± 0.72*
Lee et al.	Korea	Cross-sectional	CAD	60	129	-	-	62.1±9.8	50.3±7.6	84.2± 83.1*	89.6± 65.8*
Liu et al.	China	Case-control	AMI	113	105	62	59	61 (54−68)^#^	60(51.5−66)^#^	88.71 (86.28–92.94)^#^	65.72 (62.71–67.87)^#^
Kim et al.	Korea	Cross-sectional	CAD without diabetes	30	30	70	76.7	60.5±11	58.5±10.4	277.1 (155.6−476.6)^#^	141.7 (73.9–180.9)#
			CAD with diabetes	30	30	80	30	61.7±11	63.1±10.4	278.5 (190.5−875.1)^#^	224.5 (146.8–337.4)^#^
Shen et al.	China	Cross-sectional	CAD	136	47	72.06	48.9	68.2±9.9	64.6±9.9	277.8 (171.1−406.6)^#^	256.1 (150.6–351.9)^#^
Sinha et al.	India	Cross-sectional	SAP	70	40	71	60	65.73±10.63	59.65±10.29	166.599± 94.49791*	54.13±48.467*
Nar et al.	Turkey	Cross-sectional	SAP	78	40	74	53	63.29±10.79	58.88±11.49	101.18±141.62*	47.93±58.74*
Miyazaki et al.	Japan	Cross-sectional	CAD	224	193	76	62	70±10	66±11	26(0–86.1)^#^	25.9 (0–105.3)^#^

CAD coronary artery disease, FGF21 fibroblast growth factor 21, AMI acute myocardial infarction, ACS acute coronary syndrome, CHD coronary heart disease, MI myocardial infarction, SAP stable angina pectoris, STEMI ST-segment elevation myocardial infarction, UAP unstable angina pectoris

*Mean ± standard deviation

# Median (interquartile range)

**The value was originally reported as natural logarithm (ln) in the study and was converted using an exact black-transformation formula

The studies were conducted in five countries: eight in China [7, 8, 19, 21, 22, 24, 26, 27], two in Turkey [12, 18], two in Korea [10, 23], one in India [13], and one in Japan [25]. In terms of study design, four were case–control studies [18, 19, 21, 24], seven were cross-sectional studies [7, 8, 10, 12, 13, 23, 25], and three were prospective cohort studies [22, 26, 27].

Based on the Newcastle–Ottawa Scale (NOS), all cohort and case–control studies were rated as high quality. The cross-sectional studies were also considered high quality according to the Joanna Briggs Institute (JBI) critical appraisal checklist (Supplementary Table 1).

### Meta-analysis results

#### Association between FGF21 and CAD

**Fig. 2:**
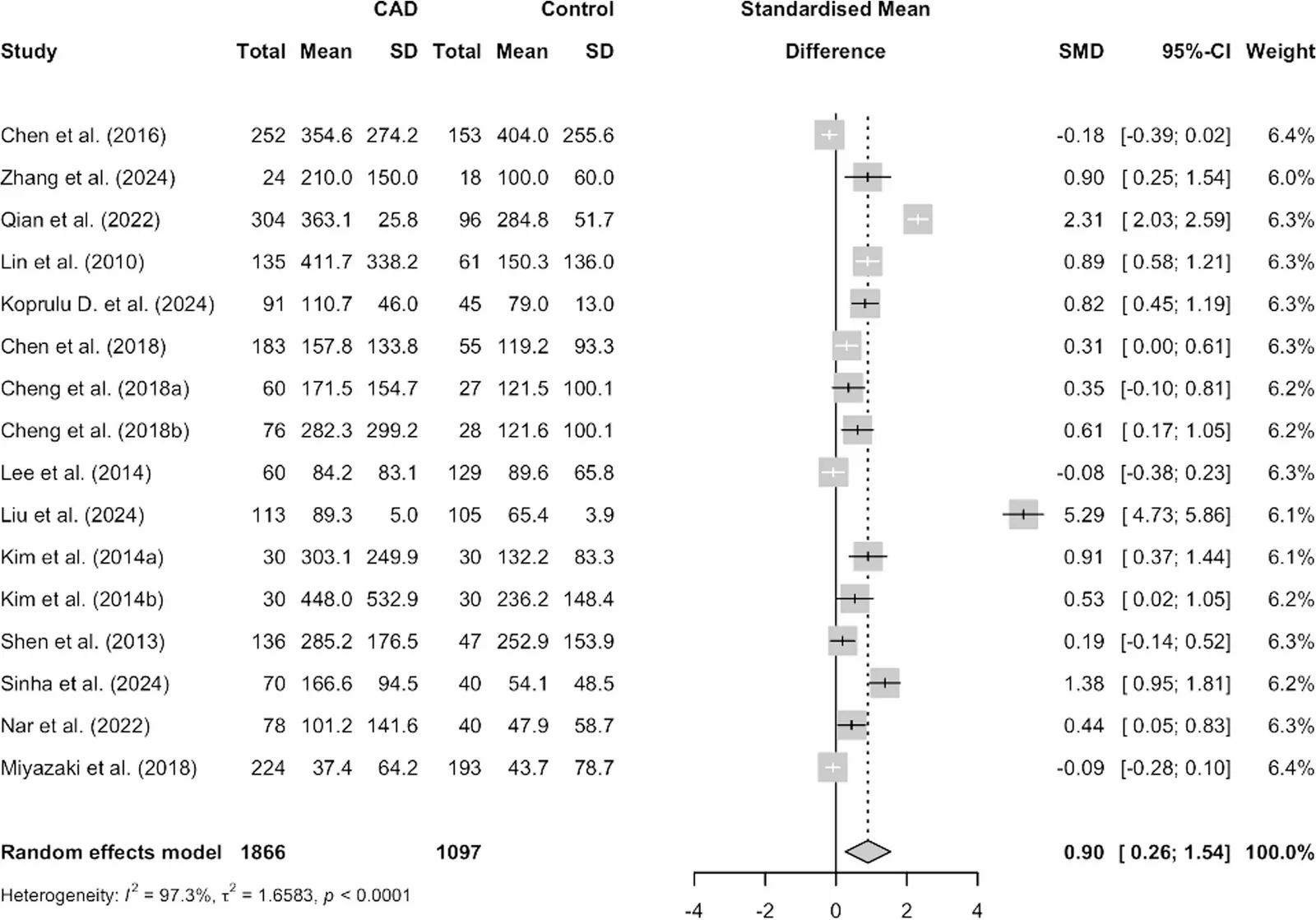
Forest plot showing serum FGF21 concentrations in individuals with and without CAD. Mean values represent the average serum FGF21 levels in each group. CAD, coronary artery disease; SD, standard deviation; SMD, standardized mean difference; CI, confidence interval

A meta-analysis was conducted to compare serum FGF21 levels between patients diagnosed with CAD and non-CAD controls using the standardized mean difference (SMD) as the measure of effect size. The pooled analysis performed under a random-effects model demonstrated an overall effect size of SMD = 0.90 (95% CI: 0.26–1.54; *p* = 0.0056). These findings indicate that serum FGF21 levels were significantly higher in patients with CAD compared to the control group. Detailed estimates of the effect size and corresponding confidence intervals are provided in Fig. 2.

Between-study heterogeneity was assessed using Cochran’s Q test and the I² statistic, revealing a very high degree of heterogeneity (Q = 561.16; df = 15; *p* < 0.0001; I² = 97.3%). This substantial heterogeneity suggests considerable variability across studies and warrants cautious interpretation of the pooled results. Accordingly, all analyses were performed using a random-effects model. The heterogeneity statistics are also summarized in Fig. 2.

#### Subgroup analyses

##### Subgroup analysis according to the percentage of diabetes in the patient and control groups

**Fig. 3:**
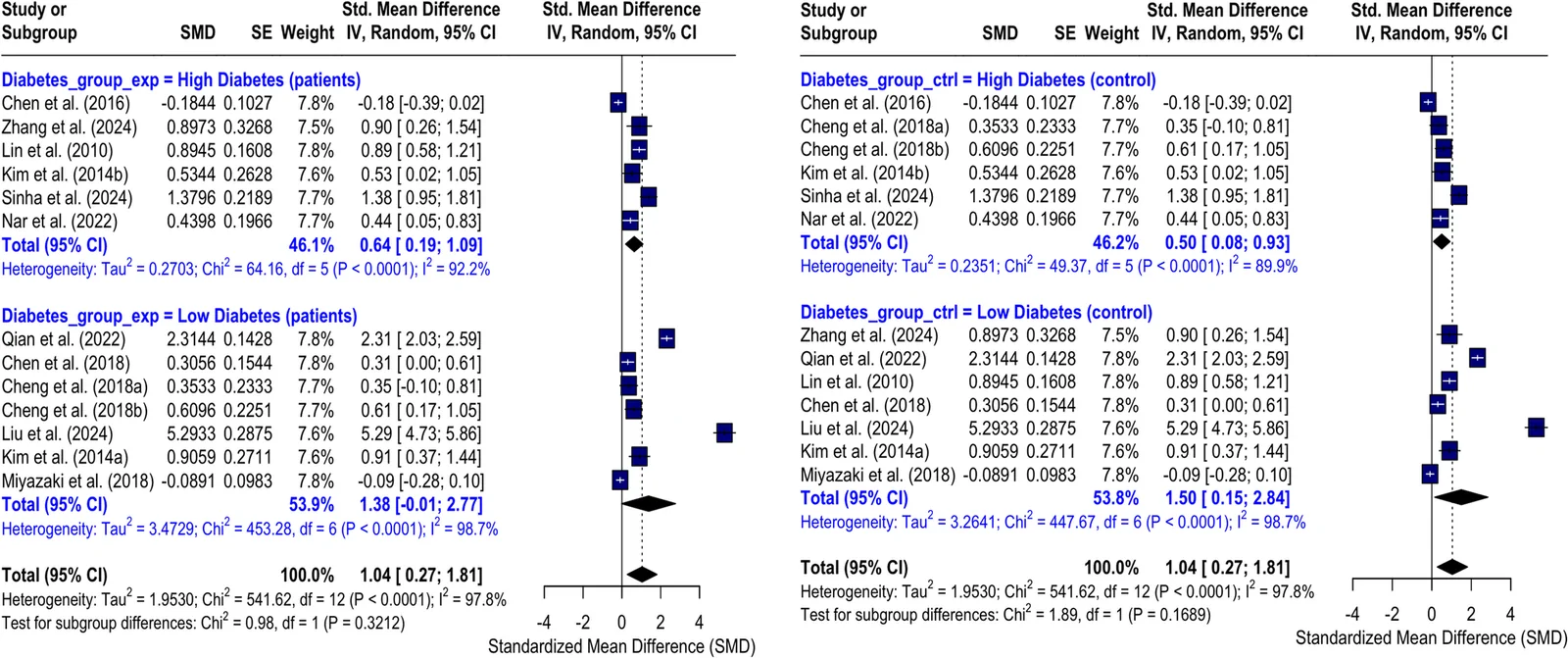
Forest plot of subgroup analysis according to diabetes prevalence: **(a)** random-effects subgroup analysis stratified by diabetes prevalence in the patient group; **(b)** random-effects subgroup analysis stratified by diabetes prevalence in the control group. *SMD* standardized mean difference, *SE* standard error, *CI* confidence interval

We performed a subgroup analysis according to diabetes prevalence. In the patient group, studies with a high diabetes prevalence showed a SMD of 0.64 (95% CI: 0.19–1.09), which was statistically significant. Although studies with low diabetes prevalence demonstrated a higher pooled SMD (1.38), the confidence interval crossed zero, indicating no statistical significance. The test for subgroup differences was also not significant (Q = 0.98, *p* = 0.32) (Fig. 3a).

A similar pattern was observed in the control group. The SMD was 0.50 (95% CI: 0.08–0.93) in studies with a high diabetes prevalence and 1.50 (95% CI: 0.15–2.84) in those with a low diabetes prevalence. Although the effect size appeared greater in the low-prevalence subgroup, the difference between subgroups was not statistically significant (Q = 1.89, *p* = 0.1689) (Fig. 3b).

##### Subgroup analysis by disease phase

**Fig. 4:**
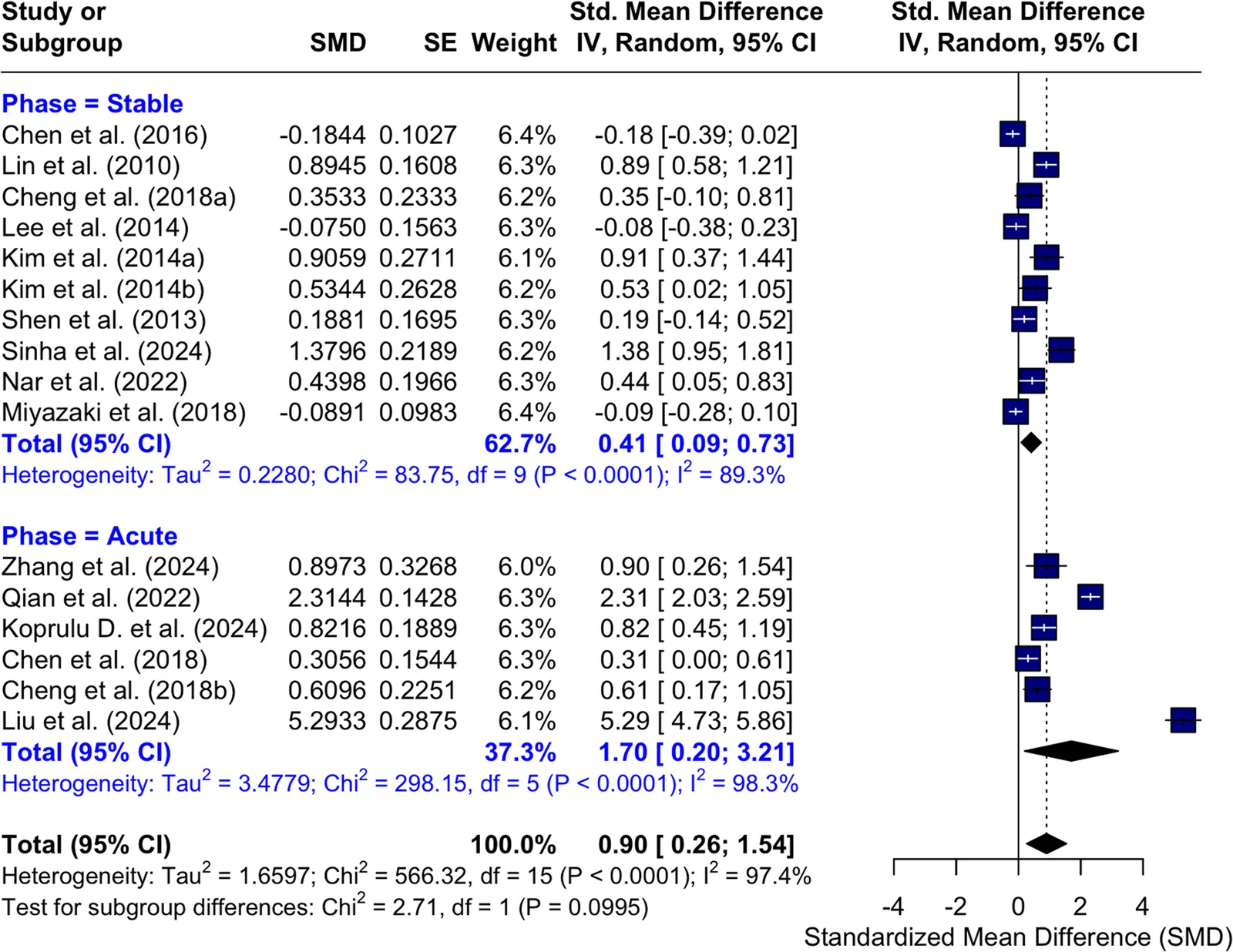
Forest plot of random-effects subgroup analysis according to disease phase. *SMD* standardized mean difference, *SE* standard error, *CI* confidence interval

Subgroup analyses stratified by disease phase demonstrated variation in the magnitude of FGF21 elevation between acute and stable conditions. Among the six studies including patients in the acute phase, the pooled effect size was large and statistically significant (SMD = 1.70; 95% CI: 0.20–3.21), with very high heterogeneity (I² = 98.3%). In contrast, the ten datasets representing the stable phase showed a smaller but still statistically significant pooled effect size (SMD = 0.41; 95% CI: 0.09–0.73). Although heterogeneity in this subgroup was lower than in the acute phase, it remained substantial (I² = 89.3%). The test for subgroup differences did not demonstrate a statistically significant difference between acute and stable phases (Q = 2.71; *p* = 0.0995) (Fig. 4).

#### Meta-regression

##### Univariate meta-regression

Univariate meta-regression analyses were performed to assess whether study-level metabolic differences could account for the observed heterogeneity. None of the examined parameters were statistically significant. The estimated standardized mean differences (SMDs) were 1.18 (SE = 1.59, 95% CI: −1.92 to 4.29, *p* = 0.4547), − 0.07 (SE = 0.39, 95% CI: −0.82 to 0.69, *p* = 0.866), − 0.19 (SE = 0.53, 95% CI: −1.22 to 0.85, *p* = 0.7213), and 0.09 (SE = 0.15, 95% CI: −0.20 to 0.39, *p* = 0.535), corresponding to LDL, HDL, BMI, and fasting glucose, respectively.

Furthermore, R² values were 0% for all models, indicating that none of the covariates accounted for any portion of the heterogeneity. These results suggest that differences in these metabolic parameters across studies do not significantly explain variability in FGF21 levels.

##### Multivariate meta-regression

A multivariable meta-regression was performed to explore sources of heterogeneity in FGF21 levels, including differences in glucose, BMI, and HDL between CAD patients and controls (k = 9). The model explained 84.6% of the heterogeneity (τ² = 0.0192, I² = 39.03%), with BMI (β = 0.44, SE = 0.11, *p* < 0.0001) and HDL (β = 0.16, SE = 0.08, *p* = 0.0477) as significant independent predictors, while glucose was not (β = −0.067, SE = 0.10, *p* = 0.50). Six datasets were excluded due to missing glucose or BMI values, and omission of studies with high effect sizes (e.g., Liu and Qian) may have influenced the estimates (Supplementary Fig. 1). Results should be interpreted cautiously given the limited number of studies and potential selection bias.

#### Sensitivity analysis

**Fig. 5:**
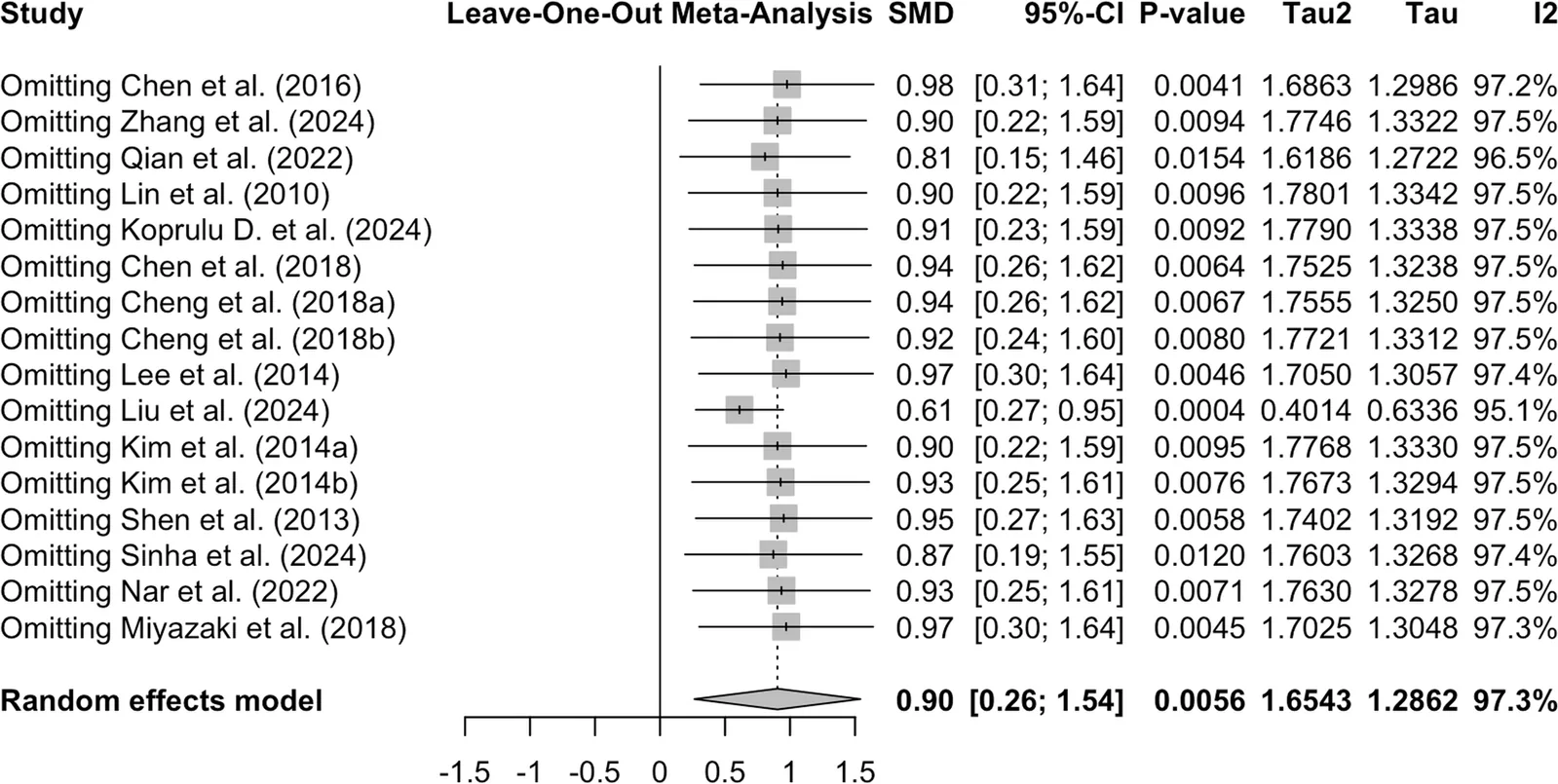
Influence analysis showing pooled effect estimates after sequential omission of each study. *SMD* standardized mean difference, *CI* confidence interval

Leave-one-out sensitivity analysis showed that exclusion of most individual studies did not materially change the pooled effect size or heterogeneity. However, the removal of the study by Liu et al. reduced the pooled effect size from 0.90 to 0.61 and decreased heterogeneity (τ²: 1.66 to 0.40; I²: 97.3% to 95.1%), indicating that this study had a substantial influence on the overall estimate (Fig. 5).

Despite its exclusion, FGF21 levels remained significantly higher in patients with CAD compared with controls (SMD = 0.61, 95% CI: 0.27–0.95; *p* = 0.0004). Heterogeneity, however, remained high (I² ≈ 95%) after removal of the study (Supplementary Fig. 2).

#### Publication bias

**Fig. 6:**
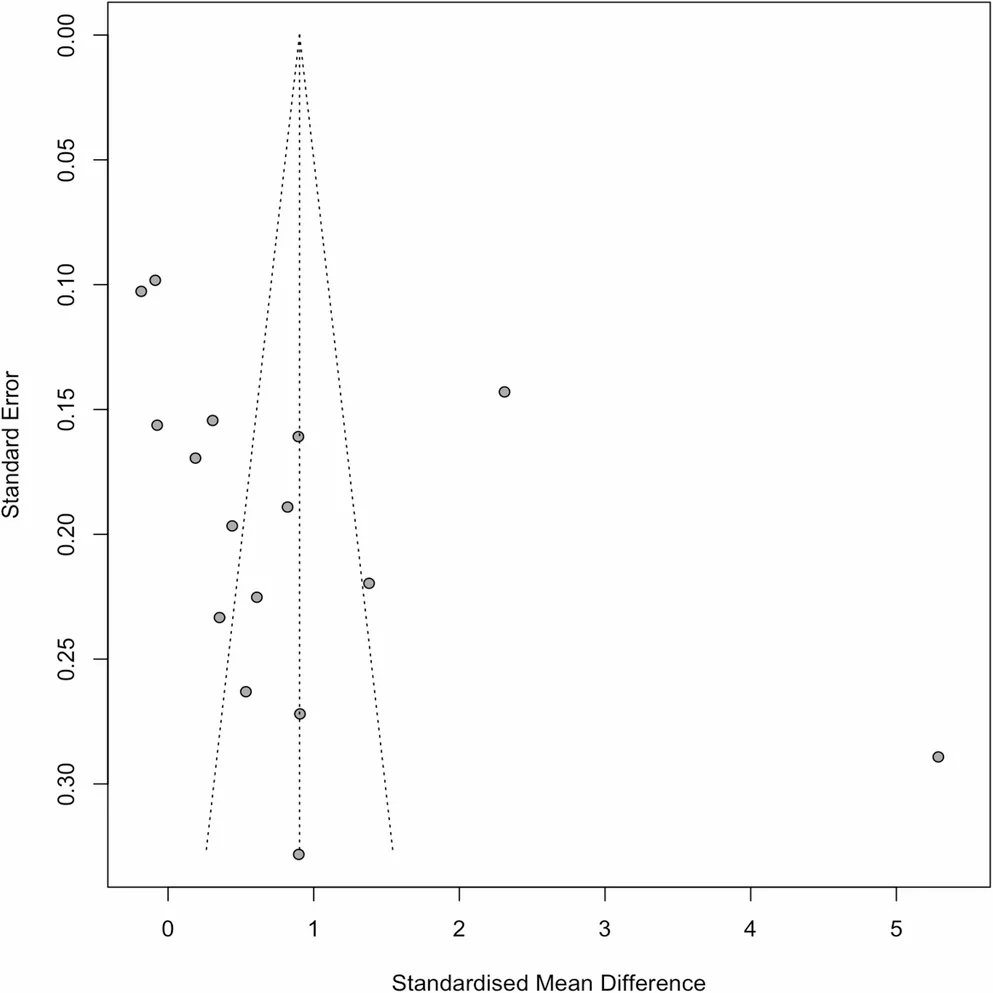
Funnel plot of publication bias

Publication bias was assessed using visual inspection of the funnel plot, Egger’s regression test, and Begg and Mazumdar’s rank correlation test. Egger’s test showed borderline evidence of asymmetry (t = 2.15, *p* = 0.0498), whereas Begg’s test did not (z = 1.89, *p* = 0.0586). Visual inspection of the funnel plot revealed no clear asymmetry, and trim-and-fill analysis did not identify any potentially missing studies. Overall, these complementary assessments provide no strong evidence that publication bias influenced the primary meta-analytic finding (Fig. 6).

## Discussion

CAD remains one of the leading causes of cardiovascular morbidity and mortality worldwide. Identifying reliable metabolic biomarkers associated with CAD may improve our understanding of disease pathophysiology and risk stratification [2].

FGF21 is a key endocrine regulator of glucose and lipid metabolism [28]. Circulating FGF21 is elevated in obesity and further increased in obese individuals with T2D [29]. Cardiomyocytes can also secrete FGF21, mediating autocrine/paracrine effects and exerting cardioprotective actions via cardiac fibroblast growth factor receptor (FGFR) pathways, reducing lipotoxicity, oxidative stress, and inflammation [30–32].

Several studies have reported higher circulating FGF21 levels in patients with CAD. Metabolic disturbances may induce a state of FGF21 resistance, explaining elevated FGF21 in cardiovascular conditions such as ACS and atherosclerosis [31]. Patients with angiographically confirmed CAD show higher fasting FGF21 levels compared with non-CAD individuals [33]. Lee et al. reported no significant difference in circulating FGF21 between CAD and non-CAD participants when groups were carefully matched for BMI, age, inflammatory markers, and lipid profiles [10]. These findings suggest that elevated FGF21 in CAD likely reflects a compensatory response to metabolic stress and a potential state of FGF21 resistance [19].

The pooled results demonstrated that circulating FGF21 concentrations were significantly higher in patients with CAD than in non-CAD controls (SMD = 0.90, 95% CI: 0.26–1.54; *p* = 0.0056). However, heterogeneity across studies was substantial (I² = 97.3%), suggesting that the findings should be interpreted with caution. This heterogeneity likely reflects differences in metabolic characteristics among study populations. Atherosclerotic cardiovascular disease, including CAD, is strongly associated with metabolic abnormalities characterized by dysregulated glucose and lipid metabolism. Elevated FGF21 levels in CAD may therefore reflect a compensatory response to metabolic stress [34]. However, the specific metabolic determinants underlying FGF21 elevations in CAD remain unclear. Lee et al. [10] observed no difference in FGF21 between CAD and control groups when BMI was strictly matched. In contrast, Lin et al. [7] reported that FGF21 was three times higher in CHD patients compared to controls even after adjusting for BMI. Similarly, while some studies have demonstrated a positive correlation between FGF21 and fasting glucose levels in CAD patients [7], others have not observed a significant association [10, 34, 35].

Our meta-regression showed that inter-study variability in FGF21 effect sizes was largely explained by cardio-metabolic parameters. A model including BMI, HDL, and glucose accounted for ~ 85% of heterogeneity, with BMI and HDL as independent determinants, while fasting glucose was not significant. Importantly, explaining such a large proportion of between-study heterogeneity strengthens the interpretation of the pooled findings and highlights the central role of metabolic characteristics. Elevated FGF21 in CAD appears more closely associated with adiposity and dyslipidemia than with glucose metabolism, consistent with previous reports [10, 34, 35].

Given that diabetes is known to raise FGF21 levels [36], it could also contribute to heterogeneity across studies. To explore this, we performed subgroup analyses based on diabetes prevalence in both patient and control groups. No statistically significant differences were observed between subgroups. Although studies with lower diabetes prevalence in both groups showed larger standardized mean differences in FGF21 levels, these differences did not reach statistical significance. Therefore, diabetes prevalence does not appear to be a major contributor to the observed heterogeneity. Importantly, FGF21 levels remained elevated in patients regardless of diabetes status, suggesting a consistent association between CAD and circulating FGF21 levels rather than a diabetes-specific effect. This observation is consistent with findings from Lin et al., who reported that CAD patients without diabetes still exhibit significantly higher FGF21 levels compared with controls [7], as well as with Cheng et al., who observed no significant difference in FGF21 concentrations between diabetic and non-diabetic patients with unstable angina [19].

CAD encompasses several clinical entities, including stable angina, ACS, and silent myocardial ischemia, with ACS typically presenting as unstable angina or myocardial infarction [37]. Acute coronary events involve metabolic and inflammatory stress, which may stimulate FGF21 production. To evaluate whether disease phase influences circulating FGF21 levels, we performed a subgroup analysis comparing acute and stable CAD. Although the difference between subgroups was not statistically significant, the pooled effect size in acute CAD was approximately four times greater than that in stable CAD, which may indicate higher FGF21 levels during acute coronary events. Consistent with this, several studies have reported elevated FGF21 in acute presentations, with patients experiencing unstable angina or ST-elevation myocardial infarction showing higher levels than those with stable angina [19, 38].

Overall, circulating FGF21 levels appear to be significantly increased in patients with CAD. However, additional well-designed studies reporting comprehensive metabolic parameters alongside FGF21 levels are needed to clarify underlying mechanisms.

This meta-analysis has several strengths. To our knowledge, it is the first quantitative synthesis of circulating FGF21 in CAD. In addition to pooled analysis, meta-regression explored heterogeneity related to key cardio-metabolic parameters (BMI, HDL, and fasting glucose), while subgroup analyses by diabetes prevalence and CAD presentation (acute vs. stable) clarified determinants of FGF21 elevation. These approaches improve interpretability and clarify the metabolic context of FGF21 in CAD.

Publication bias was evaluated using funnel plot and statistical tests. Egger’s test suggested borderline asymmetry, but Begg’s test and visual inspection of the plot showed no clear bias. Furthermore, trim-and-fill analysis did not identify any potentially missing studies. Overall, analyses suggest that publication bias is unlikely to have substantially influenced the overall findings. Nevertheless, given the relatively limited number of included studies, the possibility of undetected publication bias cannot be completely excluded.

The present meta-analysis has several limitations that should be considered when interpreting the findings. **(a)** The included studies covered different CAD types (SAP, UAP, STEMI, and NSTEMI), which may contribute to heterogeneity and limit subtype-specific generalizability. (**b)** Important indicators of glucose metabolism, such as insulin, HOMA-IR, and HbA1c, were reported in few studies, preventing a comprehensive evaluation of FGF21–glucose relationships. **(c)** Most of the included studies were conducted in Chinese populations, limiting ethnic generalizability. **(d)** Some original data, particularly those reported as medians with interquartile ranges, had to be transformed for inclusion in the meta-analysis; potential skewness in the data distribution may have affected the accuracy of these estimates. **(e)** The relatively small number of included studies (14 studies with 16 datasets) and fewer datasets for meta-regression (9) and diabetes subgroup analyses (13) may reduce statistical power and robustness. (**f)** Finally, although this meta-analysis was conducted in accordance with PRISMA guidelines, it was not prospectively registered, leaving residual risk of bias. Future large-scale studies including diverse populations with comprehensive metabolic parameters are needed, and updated meta-analyses incorporating additional prospective data will be necessary to further clarify the role of FGF21 in CAD.

In conclusion, circulating FGF21 levels are elevated in CAD patients and may be associated with metabolic disturbances—especially adiposity and dyslipidemia—rather than being exclusively related to CAD itself. Levels may rise during acute coronary events, indicating heightened metabolic and inflammatory stress. These findings suggest that FGF21 may represent a marker of metabolic stress rather than a disease-specific biomarker. This meta-analysis, through meta-regression and subgroup analyses, clarifies key determinants of elevated FGF21 in CAD, but further studies are needed to confirm its clinical utility.

## Supplementary Information

Below is the link to the electronic supplementary material.

Supplementary Material 1 (DOCX 417 KB)
