# Supplementary material for: Circulating fibroblast growth factor-21 in coronary artery disease: a systematic review and meta-analysis
Source: Ir J Med Sci. 2026 Jun 3;195(5):2707–17. doi: 10.1007/s11845-026-04472-2 (PMC13740124; doi:10.1007/s11845-026-04472-2)
Supplement: Supplementary file 1 [file 11845_2026_4472_MOESM1_ESM.docx]

**Supplementary Information**

**Title:** Circulating fibroblast growth factor-21 in coronary artery disease: A systematic review and meta-analysis

**Journal:Irish Journal of Medical Science**

**Authors:** Feryal SHAWA, Fatma IZZEDDİN, Gulsah KOC

**Corresponding author:**Gulsah KOC

**Affiliation:** Department of Medical Biology, Faculty of Medicine, Istanbul Aydin University, Istanbul, Turkey

**Email:** [gulsahkoc@aydin.edu.tr](mailto:gulsahkoc@aydin.edu.tr)

gulsahkoc@gmail.com

**Phone	: 444 1 428/52604**

**Supplementary Fig. 1** Studies included in the Multivariate Meta-regression analysis

**Supplementary Fig. 2** Forest plot of the pooled effect size after exclusion of Liu et al

**Supplementary Information**

**Title:** Circulating fibroblast growth factor-21 in coronary artery disease: A systematic review and meta-analysis

**Journal:Irish Journal of Medical Science**

**Authors:** Feryal SHAWA, Fatma IZZEDDİN, Gulsah KOC

**Corresponding author:**Gulsah KOC

**Affiliation:** Department of Medical Biology, Faculty of Medicine, Istanbul Aydin University, Istanbul, Turkey

**Email:** [gulsahkoc@aydin.edu.tr](mailto:gulsahkoc@aydin.edu.tr)

gulsahkoc@gmail.com

**Phone	: 444 1 428/52604**

Supplementary Table 1

**Table 1a** Quality assessment of Cohort studies by Newcastle–Ottawa Scale

| Included Studies | Selection | | | |  | Comparability | |  | Outcome | | | Total Scores |
| --- | --- | --- | --- | --- | --- | --- | --- | --- | --- | --- | --- | --- |
|  | A | B | C | D |  | A1 | B1 |  | A2 | B2 | C2 |  |
| Zhang (2024) | 1 | 0 | 1 | 1 |  | 1 | 1 |  | 1 | 1 | 1 | 8 |
| Qian (2022) | 1 | 0 | 1 | 1 |  | 1 | 1 |  | 1 | 1 | 1 | 8 |
| Chen (2018) | 1 | 1 | 1 | 1 |  | 1 | 1 |  | 1 | 1 | 1 | 9 |

A: Representativeness of exposed cohort. B: Selection of the non-exposed cohort. C: Ascertainment of exposure D: Demonstration that Outcome of interest was not present at start of study. A1: Important factor (If adjusted for the age, a point was assigned.) B1: Additional factor (If adjusted for any other additional factors.) A2: Assessment of outcome. B2: Was follow-up long enough for outcomes to occur. C2: Adequancy of follow up of cohorts.

**Table 1b** Quality assessment of Case-control studies by Newcastle–Ottawa Scale

| Included Studies | Selection | | | |  | Comparability | |  | Outcome | | | Total Scores |
| --- | --- | --- | --- | --- | --- | --- | --- | --- | --- | --- | --- | --- |
|  | A | B | C | D |  | A1 | B1 |  | A2 | B2 | C2 |  |
| Chen (2016) | 1 | 1 | 0 | 1 |  | 1 | 1 |  | 1 | 1 | 0 | 7 |
| Koprulu (2024) | 1 | 1 | 0 | 1 |  | 1 | 1 |  | 1 | 1 | 0 | 7 |
| Cheng (2018) | 1 | 1 | 0 | 1 |  | 1 | 1 |  | 1 | 1 | 0 | 7 |
| Liu(2024) | 1 | 1 | 0 | 1 |  | 1 | 1 |  | 1 | 1 | 0 | 7 |

A: Adequacy of case definition. B: Representativeness of the cases. C: Selection of controls. D: Definition of controls. A1: Important factor (If adjusted for the age, a point was assigned.) B1: Additional factor (If adjusted for any other additional factors.) A2: Ascertainment of exposure. B2: Same method of ascertainment for cases and controls. C2: Non-response rate.

**Table 1c** Quality assessment of Cross-section studies by using the Joanna Briggs Institute Checklist

| Included Studies | JBI quality assessment criteria’s | | | | | | | | Total score |
| --- | --- | --- | --- | --- | --- | --- | --- | --- | --- |
|  | ① | ② | ③ | ④ | ⑤ | ⑥ | ⑦ | ⑧ |  |
| Lin (2010) | Y | Y | Y | Y | Y | Y | Y | Y | 8 |
| Lee (2014) | Y | Y | Y | Y | Y | Y | Y | Y | 8 |
| Sinha (2024) | Y | Y | Y | Y | Y | U | Y | Y | 7 |
| Miyazaki (2018) | Y | Y | Y | Y | Y | U | Y | Y | 7 |
| Shen (2013) | Y | Y | Y | Y | Y | Y | Y | Y | 8 |
| Kim (2014) | Y | Y | Y | Y | Y | Y | Y | Y | 8 |
| Nar (2022) | Y | Y | Y | Y | Y | U | Y | Y | 7 |

Y: Yes, N: No, U: Unclear, NA: Not applicable. ①: Were the criteria for inclusion in the sample clearly defined? ②: Were the study subjects and the setting described in detail? ③: Was the exposure measured in a valid and reliable way? ④: Were objective, standard criteria used for measurement of the condition? ⑤: Were confounding factors identified? ⑥: Were strategies to deal with confounding factors stated? ⑦: Were the outcomes measured in a valid and reliable way? ⑧: Was appropriate statistical analysis used?

**Supplementary Information**

**Title:** Circulating fibroblast growth factor-21 in coronary artery disease: A systematic review and meta-analysis

**Journal:Irish Journal of Medical Science**

**Authors:** Feryal SHAWA, Fatma IZZEDDİN, Gulsah KOC

**Corresponding author:**Gulsah KOC

**Affiliation:** Department of Medical Biology, Faculty of Medicine, Istanbul Aydin University, Istanbul, Turkey

**Email:** [gulsahkoc@aydin.edu.tr](mailto:gulsahkoc@aydin.edu.tr)

gulsahkoc@gmail.com

**Phone	: 444 1 428/52604**

| Study | Diabetes (%) | | BMI ($kg/m^{2}$) | | HDL (mmol/L) | | LDL (mmol/L) | | Fasting Glucose (mmol/L) | |
| --- | --- | --- | --- | --- | --- | --- | --- | --- | --- | --- |
|  | CAD | Control | CAD | Control | CAD | Control | CAD | Control | CAD | Control |
| Chen et al. | 71.8 | 28.2 | 24.4±3.1 | 24.6±3.2 | 1.03±0.22 | 1.13±0.37 | 2.43±0.89 | 2.7±1.35 | 5.1±0.3 | 5.1±0.6 |
| Zhang et al. | 37.5 | 0 | 25.53±4.02 | 25.01±2.11 | 1.03±0.44 | 1.39±0.43 | 3.13±1.02 | 2.56±1.21 | - | - |
| Qian et al. | 28.3 | 10.4 | - | - | 0.88±0.21 | 0.98±0.27 | 2.14±0.8 | 2.18±0.77 | - | - |
| Lin et al. | 45.9 | 0 | 24.6±3.1 | 22.3±2.4 | 1.07±0.27 | 1.51±0.39 | 2.95±0.94 | 2.63±0.47 | 6.31±2.18 | 5.08±0.51 |
| Koprulu D. et al. | - | - | 26.49±4.52 | 25.8±3.5 | 1.10±0.39 | 1.11±0.44 | 3±1.05 | 3±1.06 | - | - |
| Chen et al. | 30.6 | 18.2 | 23.9±2.9 | 23.5±2.3 | 1±0.3 | 1.2±0.3 | 2.5±0.9 | 2.6±0.8 | 6.2±2.1 | 5.6±1.5 |
| Cheng et al. | 22.73 | 27.27 | 24.49±4.44 | 24.66±3.18 | 1.26±0.33 | 1.18±0.33 | 2.56±0.99 | 2.35±0.86 | 5.05±1.82 | 4.86±1.95 |
|  | 36.84 | 27.27 | 24.64±3.34 | 24.66±3.18 | 4.59±1.83 | 1.19±0.34 | 2.23±0.96 | 2.36±0.87 | 5.13±2.73 | 4.86±1.95 |
| Lee et al. | - | - | 24.7±3 | 24.7±3.1 | 1.28±0.35 | 1.37±0.36 | 2.63±0.74 | 2.79±0.71 | 7.35±1.92 | 5.70±1.49 |
| Liu et al. | 22 | 13 | 24.29±4.13 | 24.63±3.28 | 0.95±0.24 | 1±0.2 | 2.67±1.04 | 2.53±0.92 | - | - |
| Kim et al. | 0 | 0 | 23.9±3.4 | 22.7±3 | 1.38±0.43 | 1.38±0.40 | 2.93±0.99 | 3.14±0.84 | 6.86±1.80 | 5.62±1.03 |
|  | 100 | 100 | 25±3 | 23.5±2.8 | 1.09±0.42 | 1.21±0.36 | 2.73±1.48 | 2.86±1.21 | 12.39±5.78 | 8.86±2.45 |
| Shen et al. | - | - | 23.9±2.9 | 24±3.5 | 1.1±0.3 | 1.2±0.3 | 3±0.9 | 2.9±1 | 5.43±0.9 | 5.4±0.69 |
| Sinha et al. | 37 | 30 | - | - | 1.09±0.17 | 1.47±0.18 | 2.69±1.15 | 2.791±1.29 | 7.38±2.63 | 6.54±1.82 |
| Nar et al. | 37 | 30 | - | - | 1.08±0.30 | 1.42±0.89 | 2.74±1.15 | 2.79±1.29 | 7.04±2.73 | 6.64±1.92 |
| Miyazaki et al. | 34 | 13 | 23.7±3.3 | 23.6±4.5 | 1.32±0.34 | 1.50±0.39 | 2.97±0.83 | 2.87±0.75 | - | - |

**Supplementary Table 2** Metabolic characteristics of the studies included in the meta-analysis
